# A Network Model of the Periodic Synchronization Process in the Dynamics of Calcium Concentration in GnRH Neurons

**DOI:** 10.1186/2190-8567-3-4

**Published:** 2013-04-10

**Authors:** Maciej Krupa, Alexandre Vidal, Frédérique Clément

**Affiliations:** 1 Project-Team SISYPHE, INRIA Paris-Rocquencourt Research Centre, Domaine de Voluceau, Rocquencourt BP 105, 78153, Le Chesnay cedex, France; 2 Laboratoire Analyse et Probabilités, IBGBI, Université d’Évry-Val-d’Essonne, 23 boulevard de France, 91037, Evry cedex, France

**Keywords:** Mathematical neuroendocrinology, GnRH neurons, Calcium dynamics, Multiple time scale dynamics, Mixed-mode oscillations (MMOs), Network model, Synchronization/desynchronization, Pulsatile rhythm, Frequency control

## Abstract

Mathematical neuroendocrinology is a branch of mathematical neurosciences that is specifically interested in endocrine neurons, which have the uncommon ability of secreting neurohormones into the blood. One of the most striking features of neuroendocrine networks is their ability to exhibit very slow rhythms of neurosecretion, on the order of one or several hours. A prototypical instance is that of the pulsatile secretion pattern of GnRH (gonadotropin releasing hormone), the master hormone controlling the reproductive function, whose origin remains a puzzle issue since its discovery in the seventies. In this paper, we investigate the question of GnRH neuron synchronization on a mesoscopic scale, and study how synchronized events in calcium dynamics can arise from the average electric activity of individual neurons. We use as reference seminal experiments performed on embryonic GnRH neurons from rhesus monkeys, where calcium imaging series were recorded simultaneously in tens of neurons, and which have clearly shown the occurrence of synchronized calcium peaks associated with GnRH pulses, superposed on asynchronous, yet oscillatory individual background dynamics. We design a network model by coupling 3D individual dynamics of FitzHugh–Nagumo type. Using phase-plane analysis, we constrain the model behavior so that it meets qualitative and quantitative specifications derived from the experiments, including the precise control of the frequency of the synchronization episodes. In particular, we show how the time scales of the model can be tuned to fit the individual and synchronized time scales of the experiments. Finally, we illustrate the ability of the model to reproduce additional experimental observations, such as partial recruitment of cells within the synchronization process or the occurrence of doublets of synchronization.

## 1 Introduction

GnRH (gonadotropin releasing hormone) plays a prominent role in the control of reproductive processes in mammals. GnRH is a neurohormone released into the pituitary portal blood by hypothalamic GnRH neurons in a pulsatile manner. The pulsatile nature of this release is important for the proper functioning of the reproductive system. To date, the mechanisms behind the pulsatility are poorly understood as GnRH neurons present a significant challenge to experimental studies. They are scarce, sparsely located in the hypothalamus and interspersed with other neuronal and glial cells.

However, although GnRH neurons are sparsely located in the hypothalamus, they all have an extracerebral origin in the nasal (olfactory) placode, where they develop and from where they migrate to the brain during the development of the embryo. This feature was used in a number of studies in different species (rodents, primates, sheep) [1,2]. In particular, Terasawa et al. [3] studied cultures of pre-GnRH neurons obtained from fetuses of rhesus monkeys. It is an accepted view in the community working on placode cultures that the neurons develop in the culture in a similar way as they would in vivo [4]. Terasawa et al. [3,5] made a series of experiments, measuring GnRH release, calcium levels, and the electric activity in the cultured embryonic GnRH neurons. Remarkably, as reported in [4], placode GnRH neurons are able to release GnRH in a pulsatile manner and at frequency very close to that observed in vivo in adult animals and this process is calcium dependent. Detailed investigations of calcium dynamics revealed that calcium levels evolved in an oscillatory manner in each cell, mostly independent from cell to cell, with the exception of periodically occurring episodes of synchronization. The oscillations in the individual neurons occurred on the scale of approximately 10 min and the synchronization events roughly with the period of one hour. During the synchronization events the maximal calcium levels were typically much higher than during the independent oscillations. 

Complementary information on electric activity can be retrieved from the work of [6], as well as from another experimental approach using brain slices from mice expressing Green Fluorescent Protein (GFP) specifically in GnRH neurons [7]. These studies provide evidence of the presence of three modes of oscillation, with the two slower modes possibly related to the individual and synchronized calcium oscillations. Together with the findings of [3] this led to the following working hypothesis: The peaks of the intermediate oscillation of the electric activity coincide with the individual calcium peaks, whereas the peaks of the slowest oscillation of the electric activity coincide with the synchronized calcium peaks. Finally, since the excitation-secretion coupling mediated by calcium is well documented in other types of cells (see the discussion in [4]), we suggest the following causal sequence: increased electric activity ⟶ synchronized calcium peaks ⟶ pulse of GnRH release. 

In this article, we propose a phenomenological model that can produce patterns of oscillations consistent with the experimental results described above. On a more generic ground, our model provides a mathematical mechanism of the genesis of synchronized events superimposed on faster, individual oscillations. We introduce a three-dimensional model based on the FitzHugh–Nagumo system that reproduces the average electric activity and the intracellular calcium oscillations in individual neurons. This model has a mathematical structure that makes it possible to explain, study and control the dynamics by means of phase plane analysis. Moreover, the model can generate calcium patterns fulfilling qualitative and quantitative specifications: peak heights, baseline level, InterPeak Interval (IPI). We build the network model by introducing a network level (global) variable that mediates periodic fluctuation of excitability of the neurons, whose increase leads to episodes of electric synchronization and to calcium peaks. We show by a combination of analysis and simulations that our model can, in a robust manner, reproduce the alternation of asynchronous phases, episodes of calcium peak synchronization and postexcitatory suppression. We prove, in particular, how the time scales can be adjusted so that they agree with the individual and synchronized time scales of the experiment reported on in [3]. We also show the ability of the model to reproduce additional experimental observations, such as partial recruitment of the cells within the synchronization process and the occurrence of doublets of synchronization. 

Synchronization of coupled oscillators has been widely studied, and the ideas developed in our paper have their origin in some of these earlier works. Many studies have focused on the setting of weakly coupled oscillators, in physics; see [8] for a review, in mathematics [9,10], and in neuroscience [11]. In its simplest form, the context of such studies have been networks of coupled phase oscillators [12,13]. More general models can be reduced to coupled phase oscillators; in this reduction, the asymptotic phase of the individual oscillators, or equivalently, the foliation by isochrones, is used to derive the so-called Phase Resetting Curve, which gives rise to the coupling function [10,11]. Synchronization depends on the structure of the coupling; some of the frequently considered coupling architectures are “nearest neighbor” [10], “all-to-all” [13], and global coupling, that is coupling that depends on a global variable, e.g., the average of the phases. Examples of systems with global coupling are coupled arrays of Josephson junctions [14] and a model of the Belousov–Zhabotinsky reaction with global feedback [15]. 

More recently, some ideas have emerged on how to understand synchronization in the context of slow/fast systems using the limit of strong, rather than weak coupling; see, for example, [16]. Our model is inspired by the work of [16], who considered the so-called PING model of gamma oscillations, consisting of a population of excitatory cells and a population of interneurons, with the interneurons delivering inhibition simultaneously to all excitatory cells, thus creating a synchronizing effect. We have adapted this idea to the context of our model, creating a global variable which would have a similar, strong effect on all the members of the population, giving rise to a synchronous calcium peak. 

This article is organized as follows. In Sect. 2, we review the results of [3] and [5] in more detail, preparing the ground for the construction, analysis, and simulation of our model. In Sect. 3, we introduce and analyze the individual cell model. We additionally show how to reproduce the variability in the IPI and peak height by varying two specific parameters. In Sect. 4, we consider the coupled dynamics of a population of GnRH neurons, introduce the network model, and explain the dynamical mechanisms that underlie the emergence of the desired oscillation patterns. In particular, we show how to control the frequency of the synchronization episodes, and obtain a rigorous estimate for the simplest case. In Sect. 5, we present numerical simulations that reproduce the periodic sequence of synchronization, postexcitatory suppression and desynchronization phases. We show how to mimic partial recruitment of the cells in the synchronization episodes and how to reproduce synchronization doublets.

## 2 Intracellular Calcium Patterns in Embryonic GnRH Neurons

In this section, drawing mostly on the results of [3] and [5], we review the main qualitative and quantitative properties of intracellular calcium patterns in cultured embryonic GnRH cells. 

Calcium data in [3] and [5] were obtained by means of calcium imaging: cells were loaded with fluorochrome (fura 2) and exposed to light excitation at specific UV wavelengths. As the dye’s fluorescence properties are altered when it is bound to calcium, its relative light emission in response to different wavelengths can be used to estimate intracellular calcium concentration [17]. The data were acquired every 5 to 10 seconds during up to 170 minute periods. 

Figure 1 in [3] (http://www.jneurosci.org/content/19/14/5898/F1.expansion.html) shows time traces of intracellular calcium concentration in 10 GnRH cells. The most common patterns of variation of calcium in one cell are characterized by the following qualitative features. Each pattern consists of successive peaks characterized by a fast increase followed by a slower decrease to a baseline. Before the subsequent peak, a quiescent phase of a few minutes occurs, as either a jitter near the constant baseline calcium level or a slight and slow increase. The frequency of the oscillations in a single cell is often quite close to constant while considerable variability exists between different cells: typical patterns display InterPeak Interval (IPI) ranging between 7 and 20 minutes with an average of 8.2 minutes in [3] and 13.9 minutes in [5]. In general, the peak heights range from approximately 200 to approximately 500 nM, and the baseline ranges from 50 to 200 nM. There is a large variability in the duration of the quiescent phase: it ranges from 0 minutes (no quiescent phase) to 15 minutes. Typically, the quiescent phase duration is approximately 2/3 of the IPI. 

Figure 5 in [3] (http://www.jneurosci.org/content/19/14/5898/F5.expansion.html) shows, on the same graph, the pattern of intracellular calcium in 50 embryonic GnRH cells during 152 minutes as well as zooms of this graph over three different time intervals of 18 minute length. Each calcium pattern fits the type described above and displayed in Fig. 1 in [3]. Most of the time, the calcium patterns are independent among cells (unsynchronized, with different IPI and peak levels). 

The most striking result of [3], sometimes referred to as the Terasawa puzzle, is the existence of isolated episodes of synchronization: Almost all cells begin a peak at approximately the same time and for each cell recruited in the synchronization the height of its calcium peaks during a synchronized peak is higher than the peak heights attained outside of the synchronization periods (see Fig. 5 in [3], where three synchronized peaks are shown). These episodes of synchronization are followed by a “postexcitatory suppression” of a few minutes during which calcium levels are at the baseline in all cells. Moreover, the episodes of synchronization occur at regular intervals of nearly 60 minutes (between 59 and 61 minutes). There is also a gradual decrease in the signal amplitude (due to photobleaching) inherent in the experimental protocol and that we do not intend to capture with our modeling study. 

## 3 A Model of Intracellular Calcium Dynamics in Single GnRH Neuron

We use the excitability property of the FitzHugh–Nagumo dynamics to generate periodic oscillations of Ca that fit the qualitative pattern obtained in [3] and described in the preceding section. Moreover, the FitzHugh–Nagumo dynamics is well understood, which allows us to control the quantitative properties of the oscillatory events. We consider the following model for one neuron:

(1a) $$x^{\prime }=\tau \left(-y+4x-x^{3}-\phi_{\mathrm{fall}}(\mathrm{Ca})\right),$$

(1b) $$y^{\prime }=\tau \varepsilon k(x+a_{1}y+a_{2}),$$

(1c) $${\mathrm{Ca}}^{\prime }=\tau \varepsilon \left(\phi_{\mathrm{rise}}(x)-\frac{\mathrm{Ca}-{\mathrm{Ca}}_{\mathrm{bas}}}{\tau_{\mathrm{Ca}}}\right),$$

with

(2) $$\begin{array}{rl} \phi_{\mathrm{fall}}(\mathrm{Ca}) & =\frac{\mu \mathrm{Ca}}{\mathrm{Ca}+{\mathrm{Ca}}_{0}},\\ \phi_{\mathrm{rise}}(x) & =\frac{\lambda }{1+\exp(-\rho_{\mathrm{Ca}}(x-x_{\mathrm{on}}))}. \end{array}$$

Parameter ε>0 is assumed to be small. On the other hand, k>0 is of order 1 compared to *ε* and *τ* allows us to rescale the time variable to obtain the physical time scale of the experiments in minutes. Hence, system (1a)–(1c) is a slow–fast system with one fast variable *x* and two slow variables *y* and Ca. Variable *x* represents the electrical activity of the cell and *y* is a recovery variable as in the classical FitzHugh–Nagumo model [18]. The third variable Ca represents the intracellular calcium level. Its dynamics is mostly driven by *x* through the increasing sigmoidal function $\phi_{\mathrm{rise}}$. When $\phi_{\mathrm{rise}}$ is inactive ($\phi_{\mathrm{rise}}(x)$ close to 0), Ca decreases to a quasi steady state close to ${\mathrm{Ca}}_{\mathrm{bas}}$ which represents the baseline of the intracellular calcium level. The speed of this motion is determined by the $\tau \varepsilon /\tau_{\mathrm{Ca}}$ ratio (exponential decay rate). Ca acts as a feedback onto the *x* dynamics through the increasing function $\phi_{\mathrm{fall}}(\mathrm{Ca})$ bounded by *μ*. The effect of this coupling is to reduce the electric activity of the neuronal population in response to the rise of the calcium concentration. An analogous term is used in models of single neurons to represent the hyperpolarization of the cell membrane stimulated by calcium; see [19] for an example. We explain in the following how, in a certain range of parameter values, the cell may stay in the “hyperpolarized regime” ((x,y) to the left of the lower knee of the fast nullcline) while the values of the calcium concentration remain low.

The values of parameter $a_{i}$ are chosen according to the well-known properties of the FitzHugh–Nagumo oscillators. Hence, we take a classic cubic dynamics for the *x* dynamics. By default, we set k=1 and we assume $a_{1}$ to be negative and small, so that the *y* nullcline is steep. In the following, we set $a_{1}=-0.1$, which ensures that the *x* and *y* nullclines intersect only at one point. Parameters *μ* and ${\mathrm{Ca}}_{0}$ are positive, ensuring that $\phi_{\mathrm{fall}}(\mathrm{Ca})$ is well-defined and positive for all positive values of Ca.

### 3.1 Qualitative Study of the Single GnRH Neuron Model

Depending on the value of Ca considered as a parameter, the slow–fast FitzHugh–Nagumo oscillator (1a)–(1b) can be in an oscillatory, excitable or steady regime: 

1. Oscillatory regime: the *y* nullcline intersects the cubic *x* nullcline on its middle branch (between the two knees). This singular point is unstable and the system displays a globally attractive limit cycle of relaxation type.

2. Excitable regime: the singular point lies on either the left or the right branch close to the knee. The excitability of the system is then characterized by the following property. Let us consider the stable singular point lying on the left branch of the cubic near the left knee as initial condition. Then a small perturbation of this initial condition introduced by increasing *x* and/or decreasing *y* implies a large excursion of the orbit near the right branch of the cubic toward the right knee and back to the vicinity of the left branch before asymptotically reaching the singular point.

3. Steady regime: the singular point lies on either the left or the right branch far away from the knees: the singular point is then stable and attracts any orbit of (1a)–(1b). The perturbation from the steady state has to be large enough to bring about a large excursion in the phase portrait.

 Let us recall that the transition between the excitable state and the oscillatory regime that occurs in a very narrow interval of Ca values is the well-known canard phenomenon, leading to the existence of small attractive limit cycle following the middle branch of the cubic for a while [20]. When considering the 3D model, the periodic exploration of the regions corresponding to oscillatory regime and excitable regime of subsystem (1a)–(1b) may produce mixed-mode oscillations (MMOs). We will use this feature to reproduce the quiescent phase in the generated Ca pattern.

MMOs are a class of complex oscillations occurring in excitable systems and in particular in models of action potential generation in neurons, see [21] for a review. In this work, we will take advantage of the fact that MMO dynamics can reproduce the features of the individual calcium oscillations and that the passage between different types of MMOs can be easily controlled, especially in systems where MMOs arise via the mechanism of slow passage through a canard explosion [22,23]. 

**Fig. 1:**
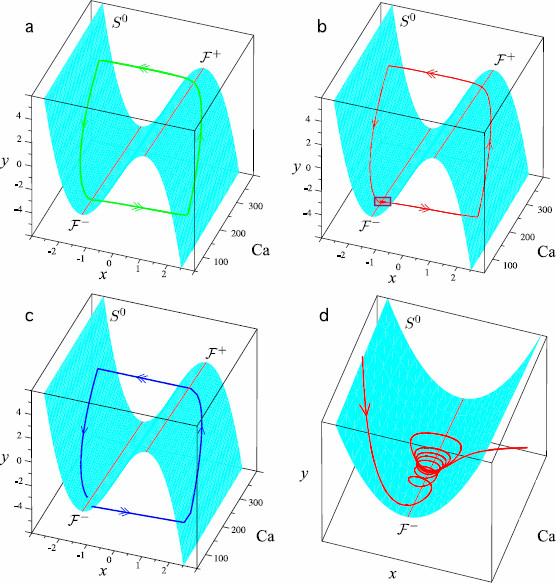
Different types of system (1a)–(1c) orbits according to the value of *μ*. In each *panel*, the *cyan surface* represents the *x* nullcline $S^{0}$ whose folds $F^{\pm }$ are represented by *red lines*. **a** Attractive periodic orbit without small oscillations near the fold $F^{-}$. This type of orbit is obtained for small values of *μ*. **b** Attractive MMO limit cycle with small oscillations near the fold $F^{-}$. This type of orbit is obtained for an interval of *μ* values. **c** Orbit that, after a transient excursion in the phase portrait, tends to the attractive singular point of system (1a)–(1c) lying on the left sheet of $S^{0}$. This type of orbit is obtained for large value of *μ*. **d** is the zoom of the *purple box* of **b** and shows a magnified view of the small oscillations of the orbit

System (1a)–(1c) is a slow–fast system with one fast and two slow variables. To describe the dynamical mechanisms underlying the behavior of the system, we introduce the following notations. The critical manifold $S^{0}$ (or *x* nullcline), given by

(3) $$y=4x-x^{3}-\phi_{\mathrm{fall}}(\mathrm{Ca}),$$

is an S-shaped surface embedding two fold lines $F^{-}$, contained in the half-space x<0, and $F^{+}$, contained in the half-space x>0. The fold lines split $S^{0}$ into three parts (see Fig. 1): the left and right sheets contained entirely in the half-spaces x<0 and x>0, respectively, and a middle sheet. The *y* nullcline, defined by

(4) $$a_{0}x+a_{1}y+a_{2}=0,$$

is a plane that crosses $F^{-}$ for a given value ${\mathrm{Ca}}_{f}$ of Ca. The Ca nullcline is an attractive surface for the Ca dynamics and is defined by

(5) $$\mathrm{Ca}=\tau_{\mathrm{Ca}}\phi_{\mathrm{rise}}(x)+{\mathrm{Ca}}_{\mathrm{bas}}.$$

The right-hand side of (5) is, like $\phi_{\mathrm{rise}}$, a smooth sigmoidal function of *x*. 

We now describe the typical interactions between the state variables, starting from a low level of Ca (i.e., close to ${\mathrm{Ca}}_{\mathrm{bas}}$) and a pair (x,y) such that (x,y,Ca) lies just below $F^{-}$. Under the influence of the fast dynamics, the current point (x,y,Ca) quickly reaches the right sheet of $S^{0}$, so that *x* and $\tau_{\mathrm{Ca}}\phi_{\mathrm{rise}}(x)$ quickly increase. Consequently, Ca increases while the current point moves up along the right sheet of $S^{0}$ toward $F^{+}$. Then, once the current point has arrived above $F^{+}$, it quickly comes back near the left sheet of $S^{0}$ under the influence of the fast dynamics; variable *x* quickly decreases as well as the term $\tau_{\mathrm{Ca}}\phi_{\mathrm{rise}}(x)$ (which becomes almost zero). The current point, driven by the slow dynamics, moves down along the left sheet of $S^{0}$ and Ca decreases eventually down to ${\mathrm{Ca}}_{\mathrm{bas}}$. Then several situations may occur depending mainly on the value of *μ* and related to the regime of system (1a)–(1b): 

A: For small values of *μ*, when the current point reaches the vicinity of $F^{-}$, system (1a)–(1b) is in the oscillatory regime. As a consequence, the current point directly and quickly reaches the right sheet of $S^{0}$, and the behavior described above repeats immediately. An example of such an orbit is represented in panel (a) of Fig. 1.

B: For an interval of values of *μ*, system (1a)–(1b) is in the excitable regime when Ca approaches ${\mathrm{Ca}}_{\mathrm{bas}}$. Then (x,y) reaches the vicinity of the singular point of (1a)–(1b) close to the left knee. Ca keeps decreasing until the current point is very close to the attractive surface given by (5). Consequently, system (1a)–(1b) passes into the oscillatory regime. During this passage, the current point makes small oscillations around the fold $F^{-}$ before it undergoes the fast transition to the right sheet and the whole motion repeats. Panel (b) of Fig. 1 represents such an orbit and panel (d) displays a magnified view of the small oscillations.

C: For large values of *μ*, system (1a)–(1b) remains permanently in the steady regime. Hence, after an excursion in the phase space, the current point reaches the attractive singular point and remains in its vicinity. Consequently, the corresponding Ca trace has one peak and remains close to the baseline afterward. Panel (c) of Fig. 1 represents such an orbit.

It is worth noticing that the interval of *μ* values corresponding to the second case depends on the other parameters, particularly on the time scale parameters *ε* and *k*. To fix the idea, we consider a particular set of parameter values given in Table 1. With these values, cases A, B, and C correspond to μ<2.26, μ∈[2.26,2.45] and μ>2.45, respectively. 

**Table 1:** Parameter values of the single cell model (1a)–(1c)

$a_{0}=1$, *ε* = 0.06, $\tau_{\mathrm{Ca}}=2$,	$a_{1}=-0.1$, *μ* = 2.4, *λ* = 175,	$a_{2}=0.8$, ${\mathrm{Ca}}_{0}=500$, $\rho_{\mathrm{Ca}}=4.5$,	*k* = 1, ${\mathrm{Ca}}_{\mathrm{bas}}=100$, $x_{\mathrm{on}}=-0.45$,	*τ* = 37.

**Fig. 2:**
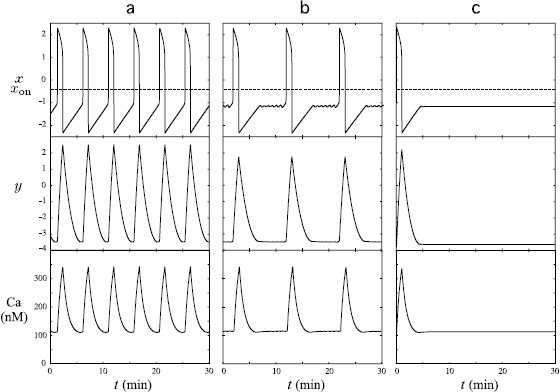
Patterns of variables *x*, *y*, and Ca generated by system (1a)–(1c) with different values of *μ*. The orbits correspond to types A, B and C described in the text and illustrated in Fig. 1. The other parameter values were chosen according to Table 1. **a** (μ=2): system (1a)–(1c) admits an attractive limit cycle of relaxation type. The increase in the calcium level is triggered by the activation of *x*, the decrease by its deactivation. The Ca pattern is oscillatory and consists of successive peaks without any quiescent phase between two successive peaks. **b** (μ=2.4): system (1a)–(1c) admits an attractive MMO limit cycle. The quiescent phase after each Ca peak is due to small oscillations of the current point near the fold $F^{-}$, which results in a slight and slow increase in Ca before the subsequent peak. The Ca pattern fulfills the average quantitative specifications provided by the experimental data. **c** (μ=3): starting from an initial condition just below the fold $F^{-}$, the Ca pattern consists of a unique peak. Afterward, the current point (x,y,Ca) tends asymptotically to a stable steady state

Figure 2 represents the *x*, *y*, and Ca patterns generated by system (1a)–(1c) with the set of parameter values given by Table 1 except for *μ* set to 2, 2.4, and 3 in panel (a), (b), and (c), respectively. The generated Ca patterns reproduce different qualitative types of calcium patterns obtained experimentally in individual cells. In panels (a) and (b), the pattern is pulsatile but in panel (c), it is composed of a single isolated peak. In panel (a), there is no quiescent phase between successive peaks. In the case of panel (b), system (1a)–(1c) admits an attractive MMO limit cycle and the small oscillations reproduce the quiescent phase of the calcium pattern at the baseline level between two successive peaks. 

**Fig. 3:**
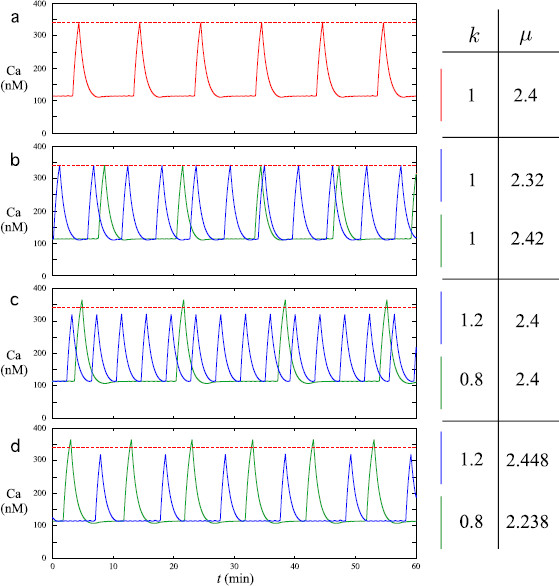
Effects of changes in the value of *μ* and *k* on the IPI and peak heights of Ca patterns generated by system (1a)–(1c). The values of *μ* and *k* corresponding to each colored pattern are given on the right of each panel. In **a**, the *red signal* is obtained with parameter values given in Table 1, the IPI equals 10 minutes and the height of the peaks is 342 nM (a *dashed red line* indicates this value in **b**, **c**, and **d** for the sake of comparison). **b** illustrates the effect of a change in *μ* on the IPIs: the *blue* (resp. *green*) *pattern* is obtained with a smaller (resp. larger) value of *μ* than the *red pattern* and has a smaller (resp. larger) IPI (7 minutes, resp. 14 minutes). **c** illustrates the effect of a change in *k* on the peak heights: an increase (resp. decrease) in the value of *k*, as in the *blue* (resp. *green*) *pattern*, implies a decrease (resp. increase) in both the height of the peaks (320 nM, resp. 365 nM) and the IPI (around 4.5 minutes, resp. 17 minutes). In **d**, we show how to hold the IPI constant (10 minutes) while obtaining a variability in the peak heights by changing *k* first (in the same way as in **c**) and then adjusting the value of *μ*

In the following, we use Table 1 as the reference set of parameter values because the generated Ca pattern (see panel (a) of Fig. 3) has the same qualitative properties as most of the patterns obtained experimentally and moreover fulfills the average quantitative specifications: the quiescence phase is twice as long as the peak duration, the peak height is around 350 nM and the IPI equals 10 minutes. 

### 3.2 Variability in Quantitative Properties of Calcium Patterns

In this section, we show how to mimic the variability of the quantitative features of calcium patterns between different cells by choosing different values for parameters of special importance: *μ* and *k*. From the above explanation, one can already understand that the precise value of *μ* prescribes the number of small oscillations of the current point near the left fold $F^{-}$ and, consequently, the duration of the quiescent phase. Since variations in *μ* do not impact much the duration of the peaks, this parameter can be considered to control the IPI. Panel (b) of Fig. 3 shows the results of a change in *μ*: an increase (resp. decrease) in *μ* value implies an increase (resp. decrease) in the IPI as shown by the green (resp. blue) pattern compared to the red one in panel (a). The range of variation in *μ* is limited by the need to produce a quiescent phase between two successive peaks in the Ca pattern.

Parameter *k* essentially tunes the time scale separation between *y* and Ca (*x* being much faster). Hence, an increase in *k* implies a shorter time for subsystem (1a)–(1b) to complete a relaxation oscillation and, consequently, a shorter time for Ca to increase and decrease back to the baseline. One can thus increase or decrease the height of the Ca peak by tuning the value of parameter *k*. Of course, a change in *k* also implies a change in the duration of the quiescence phase and, consequently, the IPI. Panel (c) of Fig. 3 shows that an increase (resp. decrease) in the value of *k* implies a decrease (resp. increase) in the height of the peaks.

The peak height and the IPI can also be chosen independently by first tuning the value of *k* and afterward the value of *μ*. Panel (d) of Fig. 3 shows the Ca patterns obtained with the same set of *k* values as in panel (c) except that the values of *μ* are chosen to balance the effect of the changes in *k* and maintain the 10 minutes IPI (μ=2.448 for the blue signal, μ=2.238 for the green one). Yet the variability in the peak heights persists.

The information on the dependence of the peak heights and the IPIs on the parameters will be used to demonstrate the ability of our network model to reproduce the experimental results. Although we will not use this in the sequel, we would like to point out that other quantitative features could be controlled by tuning other parameters of system (1a)–(1c).

## 4 Network Model

In this section, we consider the following network model of a population of GnRH neurons:

(6a) $$x_{j}^{\prime }=\tau \left(-y_{j}+4x_{j}-x_{j}^{3}-\phi_{\mathrm{fall}}({\mathrm{Ca}}_{j})\right),$$

(6b) $$y_{j}^{\prime }=\tau \varepsilon k_{j}\left(x_{j}+a_{1}y_{j}+a_{2}-\eta_{j}\phi_{\mathrm{syn}}(\sigma )\right),$$

(6c) $${\mathrm{Ca}}_{j}^{\prime }=\tau \varepsilon \left(\phi_{\mathrm{rise}}(x_{j})-\frac{{\mathrm{Ca}}_{j}-{\mathrm{Ca}}_{\mathrm{bas}}}{\tau_{\mathrm{Ca}}}\right),$$

(6d) $$\sigma^{\prime }=\tau \left(\delta \varepsilon \sigma -\gamma (\sigma -\sigma_{0})\phi_{\sigma }\left(\frac{1}{N}\sum_{i=1}^{N}{\mathrm{Ca}}_{i}-{\mathrm{Ca}}_{\mathrm{desyn}}\right)\right),$$

for j=1,…,N, with *N* the number of neurons and

(7) $$\begin{array}{rl} \phi_{\mathrm{syn}}(\sigma ) & =\frac{1}{1+\exp(-\rho_{\mathrm{syn}}(\sigma -\sigma_{\mathrm{on}}))},\\ \phi_{\sigma }(u) & =\frac{1}{1+\exp(-\rho_{\sigma }u)}. \end{array}$$

In system (6a)–(6d), function $\phi_{\sigma }$ is applied to

$$u=\frac{1}{N}\sum_{i=1}^{N}{\mathrm{Ca}}_{i}-{\mathrm{Ca}}_{\mathrm{desyn}},$$

which is the difference between the mean calcium level and the desynchronization threshold ${\mathrm{Ca}}_{\mathrm{desyn}}$. For each j=1,…,N, subsystem (6a)–(6c) (of the same type as system (1a)–(1c)) represents the activity of the *j*th cell. The values of parameters $k_{j}$ are chosen randomly using a uniform distribution in the interval [0.8,1.2] to reproduce, as explained in Sect. 3, the variability in the IPI and height of the peaks from one cell to another. The values of the parameters that have been already introduced in Sect. 3 are given in Table 1. Variable *σ* represents a global state of the network and acts on each cell through the term $\eta_{j}\phi_{\mathrm{syn}}(\sigma )$. Its dynamics consists of a very slow linear part (*ε* and *δ* are assumed to be small) and a term that depends on the level of synchronization of the network and acts as a reset mechanism when the network is sufficiently synchronized.

Note that the individual cells $\left(x_{j},y_{j},{\mathrm{Ca}}_{j}\right)$ are coupled only through variable *σ* which depends on the mean calcium concentration. This coupling is different from the one used in most synchronization studies and creates a link between calcium synchronization and higher calcium peaks. Similar global coupling arises in coupled arrays of Josephson junctions [14] as well as in a model of the Belusov–Zhabotinsky reaction with global feedback [15]. However, the specific feature of our coupling is that it is active only during very short periods when the mean calcium level is high. 

Parameter $\sigma_{0}$ plays the role of a reset value and is chosen smaller than $\sigma_{\mathrm{on}}$. Functions $\phi_{\mathrm{syn}}$ and $\phi_{\sigma }$ are increasing sigmoidal functions with inflection points at $\sigma_{\mathrm{on}}$ and 0, respectively, and are both bounded above by 1. Since they play the role of activation functions, parameters $\rho_{\mathrm{syn}}$ and $\phi_{\sigma }$ are assumed to be sufficiently large. In the limit $\rho_{\mathrm{syn}}\to +\infty$ (resp. $\rho_{\sigma }\to +\infty$), $\phi_{\mathrm{syn}}$ (resp. $\phi_{\sigma }$) converges pointwise to the following Heaviside function with activation point $\sigma_{\mathrm{on}}$ (resp. 0):

(8) $$\phi_{\mathrm{syn}}^{\infty }(\sigma )=H(\sigma -\sigma_{\mathrm{on}})=\left\{ \begin{array}{ll} 0 & \text{if }\sigma <\sigma_{\mathrm{on}},\\ 1 & \text{if }\sigma \geq \sigma_{\mathrm{on}} \end{array}\right.$$

(9) $$\left(\text{resp. }\phi_{\sigma }^{\infty }(u)=H(u)=\left\{ \begin{array}{ll} 0 & \text{if }u<0,\\ 1 & \text{if }u\geq 0 \end{array}\right.\right).$$

### 4.1 Qualitative Study of the Network Model

**Fig. 4:**
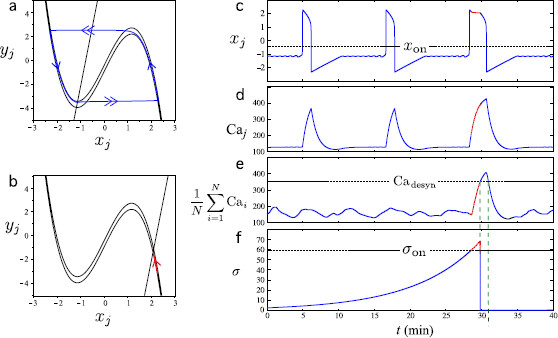
Transition of the *j*th cell of the network from independent to synchronized regime. In each *panel*, the *blue parts* correspond to the unsynchronized regime, $\sigma <\sigma_{\mathrm{on}}$ and $\phi_{\mathrm{syn}}(\sigma )≃0$, and the *red parts* to the synchronized regime, $\sigma >\sigma_{\mathrm{on}}$ and $\phi_{\mathrm{syn}}(\sigma )≃1$. **a** and **b** represent the projection of the orbit onto the plane $\left(x_{j},y_{j}\right)$ for $\sigma <\sigma_{\mathrm{on}}$ and $\sigma >\sigma_{\mathrm{on}}$, respectively, and the position of the $x_{j}$ and $y_{j}$ nullclines. The $x_{j}$ nullcline depends on ${\mathrm{Ca}}_{j}$: in each *panel*, the two *cubic curves* represent the $x_{j}$ nullcline for the minimal (*lower curve*) and maximal (*upper curve*) value taken by ${\mathrm{Ca}}_{j}$ during the corresponding regime. **c** to **f** represent the generated $x_{j}$, ${\mathrm{Ca}}_{j}$, mean calcium (among all cells) and *σ* patterns. As long as $\sigma <\sigma_{\mathrm{on}}$, each cell generates a ${\mathrm{Ca}}_{j}$ pattern with it owns rhythm: the cells are asynchronous and the mean calcium level remains low (*blue parts*). When *σ* exceeds $\sigma_{0}$ (*red parts*), $\phi_{\mathrm{syn}}(\sigma )$ is activated and the cells for which $\eta_{i}$ is large enough enter the steady regime (**b**). Hence, they produce, all almost at the same time, a higher calcium peak than in the asynchronous period of the oscillation. The mean calcium level exceeds ${\mathrm{Ca}}_{\mathrm{desyn}}$, which resets *σ* to a value close to $\sigma_{0}$

We now explain how the model can reproduce the alternation of asynchronous phases and episodes of synchronization in the case when $\phi_{\mathrm{syn}}$ and $\phi_{\sigma }$ behave as the Heaviside functions (8) and (9), respectively. We refer to Fig. 4 for a visual help on the *σ* driven transition of a particular cell of the network from the independent regime to the synchronized regime. Let us consider an initial value of *σ* just above $\sigma_{0}$. While $\sigma <\sigma_{\mathrm{on}}$, $\phi_{\mathrm{syn}}(\sigma )$ is almost zero and each cell (6a)–(6c) (for j=1,…,N) acts as described in Sect. 3. Since the values of parameters $k_{j}$ are different, each cell generates a ${\mathrm{Ca}}_{j}$ pattern with its own IPI. As a consequence, the calcium peaks are asynchronous and, as time evolves, the mean calcium level among cells, given by $\frac{1}{N}\sum_{i=1}^{N}{\mathrm{Ca}}_{i}$, remains low. As long as the mean calcium level is smaller than ${\mathrm{Ca}}_{\mathrm{desyn}}$, the second term of the *σ* dynamics is negligible. Then, since *δ* is assumed to be small, *σ* increases very slowly. This regime corresponds to the orbit in blue shown in panel (a) of Fig. 4 and the blue parts of the time series in panels (c) to (f). 

Once the mean calcium level exceeds the threshold value $\sigma_{\mathrm{on}}$, $\phi_{\mathrm{syn}}(\sigma )$ activates. Let us consider a particular cell, i.e., system (6a)–(6c) for a particular *j*. When $\phi_{\mathrm{syn}}(\sigma )$ is activated, the $y_{j}$ nullcline quickly moves to the right and, provided that $\eta_{j}$ is large enough, ends up intersecting the $x_{j}$ nullcline on its right branch as shown on panel (b) of Fig. 4. Hence, as long as $\phi_{\mathrm{syn}}(\sigma )$ is activated, the cell remains in a steady regime. The current point $\left(x_{j},y_{j}\right)$ reaches the vicinity of a singular point on the right branch and remains stationary. Therefore, the corresponding calcium level is higher than usual. Provided that sufficiently many cells are recruited in this process, the mean level quickly becomes higher than ${\mathrm{Ca}}_{\mathrm{desyn}}$. This corresponds to the red parts of the curves in Fig. 4. Then the reset term of the *σ* dynamics activates, *σ* quickly decreases, crossing back the threshold value $\sigma_{\mathrm{on}}$, to a value near $\sigma_{0}$. Consequently, $\phi_{\mathrm{syn}}(\sigma )$ is deactivated, and the whole process starts again.

It is worth noticing that all cells recruited in the event (i.e., those corresponding to a large enough value of $\eta_{j}$) were synchronized by the global variable to produce a higher calcium peak than usual. Moreover, they come back to their own pulsatile regime approximately at the same time, starting by a quiescence phase. Hence, all individual calcium levels are at the baseline for a while, before individual peaks rise again unsynchronized, which corresponds to a postexcitatory suppression.

### 4.2 Frequency of Synchronization Episodes

In Sect. 3, we have shown how to specify the parameters of individual cells to obtain the required time traces. In this section, we show how to control the network level parameters $\sigma_{0}$, $\sigma_{\mathrm{on}}$ and *δ* to obtain global synchronization with a specified frequency. In Proposition 1, we prove that the evolution of *σ* depends on the ratio $\sigma_{\mathrm{on}}/\sigma_{0}$ rather than on each of these parameters independently. Proposition 2 gives a formula for the dependence of the frequency of the synchronized peaks on *δ* and $\sigma_{\mathrm{on}}/\sigma_{0}$.

**Proposition 1***For any given*α>0, *the outputs*${\mathrm{Ca}}_{j}$*of system* (6a)–(6d) *are invariant under the change of parameter values from*$\left(\sigma_{0},\sigma_{\mathrm{on}},\rho_{\mathrm{syn}}\right)$*to*$\left(\alpha \sigma_{0},\alpha \sigma_{\mathrm{on}},\frac{\rho_{\mathrm{syn}}}{\alpha }\right)$.

*Proof* Changing the parameters from $\left(\sigma_{0},\sigma_{\mathrm{on}},\rho_{\mathrm{syn}}\right)$ to $\left(\alpha \sigma_{0},\alpha \sigma_{\mathrm{on}},\frac{\rho_{\mathrm{syn}}}{\alpha }\right)$ in system (6a)–(6d) yields

(10a) $$x_{j}^{\prime }=\tau \left(-y_{j}+4x_{j}-x_{j}^{3}-\phi_{\mathrm{fall}}({\mathrm{Ca}}_{j})\right),$$

(10b) $$y_{j}^{\prime }=\tau \varepsilon k_{j}\left(x_{j}+a_{1}y_{j}+a_{2}-\eta_{j}\bar{\phi_{\mathrm{syn}}}(\sigma )\right),$$

(10c) $${\mathrm{Ca}}_{j}^{\prime }=\tau \varepsilon \left(\phi_{\mathrm{rise}}(x_{j})-\frac{{\mathrm{Ca}}_{j}-{\mathrm{Ca}}_{\mathrm{bas}}}{\tau_{\mathrm{Ca}}}\right),$$

(10d) $$\sigma^{\prime }=\tau \left(\delta \varepsilon \sigma -\gamma (\sigma -\alpha \sigma_{0})\phi_{\sigma }\left(\frac{1}{N}\sum_{i=1}^{N}{\mathrm{Ca}}_{i}-{\mathrm{Ca}}_{\mathrm{desyn}}\right)\right),$$

where the new function $\bar{\phi_{\mathrm{syn}}}$ is given by

(11) $$\begin{array}{rl} \bar{\phi_{\mathrm{syn}}}(\sigma ) & =\frac{1}{1+\exp(-(\rho_{\mathrm{syn}}/\alpha )(\sigma -\alpha \sigma_{\mathrm{on}}))}. \end{array}$$

Changing *σ* to *ασ*, and using the relation

(12) $$\begin{array}{rl} \bar{\phi_{\mathrm{syn}}}(\alpha \sigma ) & =\phi_{\mathrm{syn}}(\sigma )=\frac{1}{1+\exp(-\rho_{\mathrm{syn}}(\sigma -\sigma_{\mathrm{on}}))}, \end{array}$$

one obtains precisely system (6a)–(6d) with former parameters $\left(\sigma_{0},\sigma_{\mathrm{on}},\rho_{\mathrm{syn}}\right)$. □

*Remark 1* As explained at the beginning of the section, the value of $\rho_{\mathrm{syn}}$ is chosen large enough so that the activation of $\phi_{\mathrm{syn}}$ is almost immediate. It is worth noticing that a change in this value, provided that it remains large (typically greater than 10), does not affect the qualitative features of the model outputs (periodic episodes of synchronization) and has a limited impact on the period between two successive episodes of synchronization. Proposition 1 shows that, for $\rho_{\mathrm{syn}}$ large enough and a fixed value of *δ*, the period of the synchronization episodes depends mainly on the ratio $\sigma_{\mathrm{on}}/\sigma_{0}$. On the other hand, the value of these parameters can be chosen arbitrarily (provided that $\sigma_{0}<\sigma_{\mathrm{on}}$) and the synchronization period can be adjusted by choosing the value of *δ* as proved in Proposition 2.

**Proposition 2***In the case*$\rho_{\sigma }=\infty$, *forγlarge enough relative toεandδ*, *the period between two successive episodes of synchronization in system* (6a)–(6d) *is approximated by*

(13) $$T_{\mathrm{syn}}=\frac{1}{\tau \varepsilon \delta }\ln\frac{\sigma_{\mathrm{on}}}{\sigma_{0}}.$$

*Remark 2* If the values of all the parameters of system (6a)–(6d), except *δ*, are fixed, we can adjust the synchronization period in the ${\mathrm{Ca}}_{j}$ pattern to any value $T_{\mathrm{syn}}>0$ by choosing

$$\delta =\frac{1}{\tau \varepsilon T_{\mathrm{syn}}}\ln\frac{\sigma_{\mathrm{on}}}{\sigma_{0}}$$

*Proof of Proposition 2* As explained above, each episode of synchronization results in a decrease of *σ*, under the influence of the calcium dependent part of its dynamics. For a value of *γ* large compared to *ε* and *δ*, the *σ* dynamics, in the period when $\phi_{\sigma }$ is active, is much faster than the ${\mathrm{Ca}}_{j}$ dynamics. Since $\rho_{\sigma }=\infty$, *σ* decreases quickly down to a value very close to the singular point $\bar{\sigma }$ of its dynamics defined by

$$\delta \varepsilon \bar{\sigma }-\gamma (\bar{\sigma }-\sigma_{0})=0$$

i.e.,

$$\bar{\sigma }=\frac{\gamma \sigma_{0}}{\gamma -\delta \varepsilon }=\sigma_{0}+O\left(\frac{\varepsilon \delta }{\gamma }\right).$$

Hence, the time $T_{\mathrm{syn}}$ between two successive synchronization episodes is approximately given by the time needed for *σ* to increase from $\sigma_{0}$ up to $\sigma_{\mathrm{on}}$. Let us recall that the cells are asynchronous during this phase and

$$\phi_{\sigma }^{\infty }\left(\frac{1}{N}\sum_{i=1}^{N}{\mathrm{Ca}}_{i}-{\mathrm{Ca}}_{\mathrm{desyn}}\right)=0.$$

It follows that, for $\sigma <\sigma_{\mathrm{on}}$, the *σ* dynamics is given by its linear part: $\sigma^{\prime }=\delta \varepsilon \sigma$. By direct integration, one obtains

(14) $$\sigma_{0}\exp(\tau \varepsilon \delta T_{\mathrm{syn}})=\sigma_{\mathrm{on}}\;\Leftrightarrow \;T_{\mathrm{syn}}=\frac{1}{\tau \varepsilon \delta }\ln\frac{\sigma_{\mathrm{on}}}{\sigma_{0}}.$$

 □

## 5 Three Types of Synchronization Episodes

In this section, we first show the ability of the model described by system (6a)–(6d) to reproduce sequences of synchronization events separated by asynchronous oscillations of the individual cells. We apply the results of Propositions 1 and 2 to control the periods and patterns of both the individual oscillations and the synchronized calcium peaks. Subsequently, we consider the question of introducing heterogeneity in the values of parameters $\eta_{j}$, since they modulate the influence of the global variable *σ* on the *j*th cell. This heterogeneity allows us to account for the additional feature of partial recruitment. We also show that parameters $\eta_{j}$ can be selected so that the desynchronization mechanism is weaker, which may lead to doublets of synchronization. In Sect. 5.1, we discuss the case of full synchronization with no doublets, which corresponds to equal, sufficiently large values of $\eta_{j}$. In Sect. 5.2, to obtain partial synchronization, we introduce unequal values of $\eta_{j}$ including ones that are sufficiently small, so that the corresponding cells are not recruited by the synchronization mechanism. In Sect. 5.3, in order to obtain doublets of synchronization, we choose the values of $\eta_{j}$ so that the $x_{j}$ and $y_{j}$ nullclines intersect near the upper fold of the $x_{j}$ nullcline. In this case, we choose in addition a relatively small value of parameter *γ* to impose a slow decrease of the global variable *σ*.

### 5.1 Full Synchronization of Intracellular Calcium Peaks in a Network of GnRH Cells

In Table 2, we introduce the values of the network level parameters for system (6a)–(6d). The value δ=0.05 is obtained from equation (13) using $T_{\mathrm{syn}}=60$ min and the parameter values of Table 2. Moreover, for now, the same value is used for all parameters $\eta_{j}$, so that the effect of *σ* on each cell is the same. The value of ${\mathrm{Ca}}_{\mathrm{desyn}}$ is chosen just above the mean calcium peak height of individual ${\mathrm{Ca}}_{j}$ pattern (i.e., the one generated by the three-dimensional system (1a)–(1c) with parameter values in Table 1). This ensures that random synchronization between few cells will not interrupt the slow increase of *σ* as the mean calcium level among all cells will not exceed ${\mathrm{Ca}}_{\mathrm{desyn}}$. This happens only if a sufficient number of cells generate at the same time a greater calcium peak than usual. 

**Table 2:** Parameter values of the network model (6a)–(6d) for generating full synchronization episodes

*δ* = 0.05, $\rho_{\mathrm{syn}}=5$,	*γ* = 20, $\rho_{\sigma }=30$,	$\eta_{j}=3$, $\sigma_{\mathrm{on}}=60$,	${\mathrm{Ca}}_{\mathrm{desyn}}=350$, $\sigma_{0}=0.1$.

**Fig. 5:**
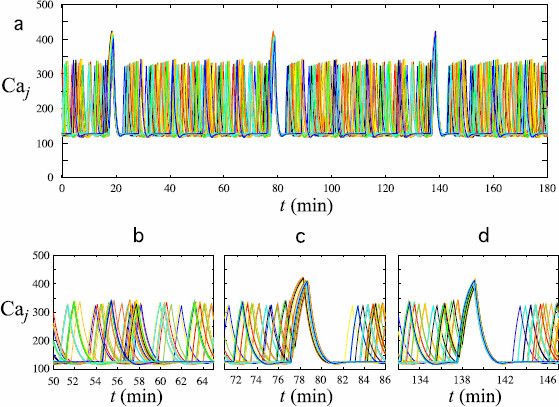
Patterns of calcium oscillations in a network of 50 GnRH neurons. *Ten different colors* have been used; each *color* is used to represent the activity of 5 individual cells. **a** represents the 50 individual ${\mathrm{Ca}}_{j}$ patterns along a 180 minute period. Note that synchronization occurred at minute 17, 78, and 139, which gives 61 minute intervals between synchronizations. The height of the synchronized peaks is larger than the height of normal peaks, and the postexcitatory suppressions are seen right after each synchronization episode. **b** displays a magnified view of the asynchronous phase occurring between successive episodes of synchronization. The ${\mathrm{Ca}}_{j}$ patterns display variability in IPI and height of the peaks between cells. Depending on the phases of each cell when the synchronization is triggered, the calcium peaks may be more or less tightly synchronized as emphasized in **c** and **d**

Panel (a) of Fig. 5 displays in the same graph the ${\mathrm{Ca}}_{j}$ patterns generated by system (6a)–(6d) with N=50 along a 180 minute interval. Outside the synchronization episodes, the oscillations are asynchronous, with each cell producing calcium peaks at its own frequency. Synchronization episodes take place every 61 minutes (at minute 17, 78, and 139). Panel (b) is a magnified view of the ${\mathrm{Ca}}_{j}$ patterns during the unsynchronized phase (over a 15 minute interval). Note that, due to the variability in the $k_{j}$ values, the heights of the calcium peaks and the IPIs differ from one cell to another. Panels (c) and (d) show magnified views of two synchronization episodes. All cells are recruited in both episodes, resulting in higher calcium peaks than usual for all cells followed by complete postexcitatory suppression. 

Note that the calcium peaks can be more or less tightly synchronized from one synchronization episode to another along the same trajectory of system (6a)–(6d). The extent of tightness can be assessed, following [4], as the length of the time interval with end points given by the time instances of the earliest and latest peak corresponding to a given synchronization event. In the synchronization episodes displayed in panels (c) and (d), this length equals 37 and 13 seconds, respectively. The variability in the tightness between different synchronization episodes is related to the maximum of the phase differences between each couple of oscillators (6a)–(6c) when the synchronization is triggered. Moreover, the time needed by each ${\mathrm{Ca}}_{j}$ to decrease back to the quiescent phase also depends on the relative positions of the $x_{j}$ and $y_{j}$ nullclines when $\phi_{\mathrm{syn}}(\sigma )$ is activated. These positions are mainly characterized by the values of parameters $\eta_{j}$. Hence, the tightness of synchronization episodes is also related to the sensitivity of each cell to the synchronization mechanism. Since the time scale differences between cells and the sensitivity to the synchronization mechanism interact in an intricate way, the precise study of the tightness of synchronization is a challenging problem.

### 5.2 Partial Recruitment

As mentioned in the preceding section, parameter $\eta_{j}$ tunes the impact of variable *σ* upon the corresponding 3D system (6a)–(6c). Hence, it represents the sensitivity of the cell to the impact of the network state. One can mimic the variability in this sensitivity among cells by choosing different values of $\eta_{j}$.

**Fig. 6:**
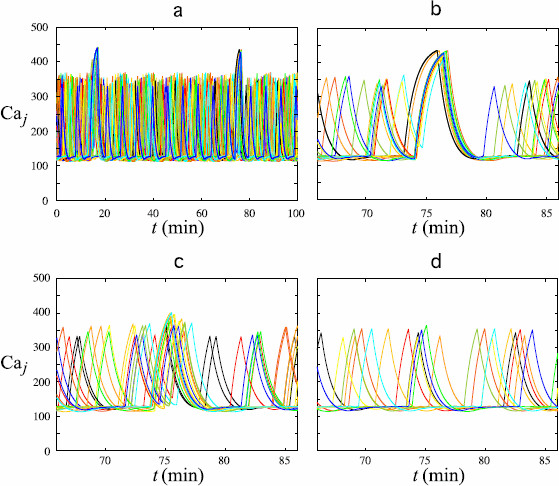
Patterns of calcium oscillations in the 50 GnRH cells of a network with various sensitivity to the synchronization process. *Ten different colors* have been used; each *color* is used to represent the activity of 5 individual cells. In **a**, the ${\mathrm{Ca}}_{j}$ patterns in all cells are shown for a period of 100 minutes. Only a part of the cells participates in the synchronization episodes at t≃17 minutes and t≃76 minutes. The *three other panels* display magnified views of the second synchronization episodes by assembling the ${\mathrm{Ca}}_{j}$ patterns in cells, which are completely recruited in this event (**b**), recruited with no significant increase in the peak level (**c**) or not recruited at all (**d**)

Panel (a) of Fig. 6 shows the ${\mathrm{Ca}}_{j}$ patterns generated by system (6a)–(6d) with parameter values given by Tables 1 and 2, with the exception of $\eta_{j}$ whose values have been chosen randomly in [0,3]. Only 20 cells with sufficiently large value of $\eta_{j}$ are completely recruited in the synchronization episodes and generate calcium peaks significantly higher than usual: their ${\mathrm{Ca}}_{j}$ patterns are assembled in panel (b). For 18 other cells (panel (c)), the ${\mathrm{Ca}}_{j}$ peaks are not significantly higher than usual, even if they are synchronized with those in panel (b). These cells corresponds to intermediate values of $\eta_{j}$. Moreover, in these ${\mathrm{Ca}}_{j}$ patterns, the last IPI before the synchronization episode is much shorter than usual, which indicates that it does not result from a random coincidence but the cells actually undergo the effect of the synchronization process. Finally, the ${\mathrm{Ca}}_{j}$ patterns of the 12 remaining cells with low values of $\eta_{j}$ (panel (d)) are not recruited by the synchronization mechanism: their peak heights are unchanged, their IPI remains constant during the synchronization episode and the calcium level can even be at the baseline. 

### 5.3 Doublets

As explained in Sect. 4, the reset mechanism of *σ* is introduced through a fast part of the dynamics activated by the synchronization of the calcium peaks. The strength of this mechanism is controlled by the value of parameter *γ* and, in contrast with a classical reset, the decrease of *σ* can be tuned by choosing the range of *γ* values. In the preceding simulations, the values of *γ* were chosen large enough (compared to *ε*) so that, through the reset mechanism, *σ* can decrease down to a value very close to $\sigma_{0}$ before the mean calcium level decreases below the threshold ${\mathrm{Ca}}_{\mathrm{desyn}}$. In the following, we show how to reproduce doubled episodes of synchronization by slowing down the *σ* decrease induced by the synchronization of calcium peaks, i.e. by choosing a smaller value of *γ*.

To ensure that, after the episode of synchronization, the mean calcium level decreases as soon as *σ* starts to decrease, we select small enough values of $\eta_{j}$. For the sake of simplicity, we consider the same value for all $\eta_{j}$, j=1,…,N, since the phenomenon of synchronization as doublet does not require variability in the cell sensitivity to synchronization. Hence, we consider the set of parameter values given in Table 3. 

**Table 3:** Parameter values of the network model (6a)–(6d) for generating synchronization as doublets

*δ* = 0.05, $\rho_{\mathrm{syn}}=5$,	*γ* = 0.3, $\rho_{\sigma }=30$,	$\eta_{j}=1.12$, $\sigma_{\mathrm{on}}=60$,	${\mathrm{Ca}}_{\mathrm{desyn}}=380$, $\sigma_{0}=0.1$.

**Fig. 7:**
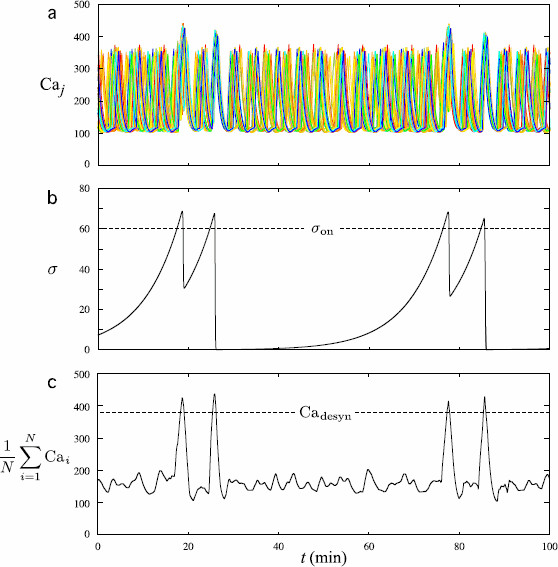
Synchronization as doublets in patterns of calcium oscillations in the 50 GnRH cells of a network. The parameter values of system (6a)–(6d) are given in Tables 1 and 3. In **a**, *ten different colors* have been used to represent the ${\mathrm{Ca}}_{j}$ patterns, hence each color is used to represent the activity of 5 individual cells. **b** represents the corresponding patterns of *σ* as well as the synchronization threshold $\sigma_{\mathrm{on}}$ (*dotted line*). **c** represents the mean calcium level $\frac{1}{N}\sum_{i=1}^{N}{\mathrm{Ca}}_{i}$ as well as the threshold ${\mathrm{Ca}}_{\mathrm{desyn}}$ (*dotted line*) which triggers the decrease of *σ*. Doublets of synchronization occur at minutes 19–26 and minutes 78–86: the two first episodes of synchronization are separated by a 60 minute period. The doublets occur because, after the first episode has been triggered, the mean calcium value stays above the threshold ${\mathrm{Ca}}_{\mathrm{desyn}}$ only during a short time and, since the *γ* value is small, *σ* starts increasing again before reaching $\sigma_{0}$ vicinity. Consequently, the subsequent synchronization episode occurs only 7 minutes afterward. It is worth noticing that the synchronization during the second episode of each doublet is tighter than during the first one. Hence, the subsequent decrease in *σ* is stronger than the preceding one, which results into a 56 minute period before the following doublet

Figure 7 represents the outputs of system (6a)–(6d) using the parameter values given in Table 3. The ${\mathrm{Ca}}_{j}$ patterns show synchronization episodes occurring as doublets. The first episode of synchronization occurs around minute 19 when *σ* becomes greater than $\sigma_{\mathrm{on}}$. The tightness of this synchronization event is quite long, around 50 seconds. Consequently, the corresponding peak in the mean calcium level is not much higher than the asynchronous peaks. When *σ* decreases below $\sigma_{\mathrm{on}}$, the mean calcium level decreases and quickly becomes smaller than ${\mathrm{Ca}}_{\mathrm{desyn}}$. Parameter *γ* is small enough so that the *σ* reset mechanism is not entirely completed: *σ* starts increasing again from a value much greater than $\sigma_{0}$. Hence, a second episode of synchronization occurs few minutes later (at minute 23) and the corresponding synchronization is tighter than the preceding one, with the calcium peaks occurring in a time interval of 25 second length. The second mean calcium peak of a synchronization doublet is thus higher than the first one. Subsequently, the time needed for the mean calcium level to decrease below ${\mathrm{Ca}}_{\mathrm{desyn}}$ is long enough for the *σ* reset mechanism to be completed. Since variable *σ* slowly increases again from a value close to $\sigma_{0}$, the subsequent asynchronous phase lasts 56 minutes. The second doublet of synchronization results from the same mechanism. 

Note that the time separating two synchronization episodes of a doublet depends strongly on the value of parameter *γ* and the tightness of synchronization of the first episode. There is a strong variability in this duration from one doublet to another in a same set of ${\mathrm{Ca}}_{j}$ patterns. Hence, reproducing a given sequence of doublet is a challenging problem. It is worth noticing that the variability in the doublets reproduced with the model is consistent with the variability observed in the experimental data [4]. 

## 6 Discussion

In this paper, we have presented a network model capable of reproducing the salient features of calcium oscillations that were observed by Terasawa and coworkers [3-5] in their experiments on GnRH neurons in placode cultures. As observed in the experiments and recovered by our model, individual cells oscillate independently, with a significant variation in the oscillatory pattern. Superimposed on the individual oscillations are synchronized events that can be described as almost simultaneous occurrences of calcium peaks, typically higher than in the absence of synchronization. Our model can reproduce these features in a very efficient way: By changing the parameters according to very simple rules, we can design the individual patterns of oscillation as well as control the frequency of the synchronization events. In addition, we can reproduce the phenomena of partial recruitment and irregularities of the synchronization patterns, for example doubled episodes of synchronization. 

To reproduce the irregularity of the individual oscillations a set of parameters controlling the individual patterns is drawn at random before each simulation. As the cells are coupled only during the synchronization episodes, the individual phases appear from the simulation to be completely ergodic. This may also be related to our choice of the parameters, corresponding to the sensitive dynamics of MMOs. Introducing even weak coupling between the cells might lead to some phase locking between the patterns, thus making the phases less irregular. This in turn could be destroyed by adding external noise. In this study, we have chosen not to include these effects.

In this work, we have used experimental specifications as a guide for finding and tuning the model. One of the main challenges of our work was to design a model pertinent to the slow and the super-slow time scales. The key idea behind reproducing the synchronization episodes was to introduce a global variable whose increase would result in a significant increase of calcium peaks and which would then be “reset” by high level of calcium. There is a parallel between this approach and the phenomenon of Calcium Induced Calcium Release (CICR), which can be understood as a self amplification of calcium release involving the depletion of intracellular calcium stores (sometimes following a depolarizing current); see [24] for more details and [1] for the available information on CICR in different types of GnRH neurons. In the model, the individual CICR mechanism is not embedded as such in the calcium dynamics of individual neurons. However, the network dynamics enables individual neurons to sustain a prolonged elevation in intracellular calcium levels at the time of the synchronized peaks, so that one can be tempted to interpret this effect as a CICR-like phenomenon. Due to the lack of precise information on the physical coupling between cultured neurons as well as on the nature of calcium stores that are effectively mobilized in embryonic neurons [25], we can only speculate on that issue. Following [26], we could conjecture that this effect is mediated through the nonneuronal cells that also exhibit calcium oscillations [5] and signal to GnRH neurons via ATP (Adenosine Triphosphate) through the ionotropic receptor P2X (a ligand-gated ion channel). The pharmacological control of ATP levels alters both synchronized calciums peaks and GnRH release. Hence, the synchronized peaks in GnRH neurons might be associated with a CICR mechanism induced by ATP inputs coming from the nonneuronal cells. 

Other works have dealt with modeling the individual dynamics of GnRH neurons, representing the activity of the membrane potential and the ionic channels, in the style of the Hodgkin–Huxley system; see [27] for a study of GnRH neurons in brain slices from adult mice. A way to link our study to microscopic modeling of this kind could be by means of developing a firing rate model that would, in a similar way as our model, accurately reproduce the slow and super-slow timescales of individual and synchronized calcium oscillations as well as could be derived from a detailed microscopic model by means of averaging. 

The main goal of this paper has been to design a model of coupled oscillators that could reproduce the experimental results of Terasawa [3] and in which we could identify the parameters controlling the most relevant features of the experimental observations, mainly the durations of the intervals separating the individual calcium peaks and synchronization events. Similar results may have been obtained by a different approach, for example by adapting a model of population spikes [28], in which episodic synchronous spikes arise due to the presence of slowly varying parameters that make the system oscillate between the regions of synchronization and asynchronous behavior. A very useful feature of our model that may be difficult to obtain in such settings is the simple dependence of both the periods of the individual calcium peaks and synchronization events on the system parameters. 

Finally, we would like to mention an aspect of the dynamics that our model was not designed to reproduce, namely a spatial structure in the patterns of spatial synchronization; see Fig. 4 in [4]. Spatial structures of calcium dynamics can be studied in continuous models; see, e.g., [29] for a study in the context of CICR. Spatial patterns registered by Terasawa and coworkers could possibly be understood in a mean field model derived from our network system. 

## List of Abbreviations

GnRH: Gonadotropin Releasing Hormone

IPI: InterPeak Interval

MMOs: Mixed-Mode Oscillations

## Competing Interests

The authors declare that they have no competing interests.

## Authors’ Contributions

FC identified the modeling issue and designed the biological specifications. MK conceived and built the model. AV improved and calibrated the model and performed the simulations. All authors participated in writing the manuscript. All authors read and approved the final manuscript.
